# Applying the Theory of Planned Behavior to predict community pharmacists’ intention to provide diabetes care

**DOI:** 10.1186/s12913-022-08788-4

**Published:** 2022-12-05

**Authors:** Dalia EL-Kaffash, Ebtisam Fetohy, Azza Mehanna

**Affiliations:** 1 Master Health Education and Behavioral Sciences, High Institute of Public Health, Alexandria University, 105 Gamal Abd EL-Nasser Street Sidi Bishr, Alexandria, 21624 Egypt; 2 Health Administration and Behavioral Sciences Department, High Institute of Public Health, Alexandria University, Alexandria, Egypt

**Keywords:** Theory of Planned Behavior, Intention, Structural equation model, Type 2 diabetes, Diabetes care, Community pharmacists

## Abstract

**Background:**

The role of the pharmacist has changed from dispensing medicines, to working with other healthcare professionals to assure appropriate medication therapy management. This study assessed community pharmacists’ intention regarding diabetes care based on the theory of planned behavior (TPB) in Alexandria, Egypt.

**Methods:**

A sample of 385 community pharmacies with one index per site (one pharmacist per pharmacy) was recruited in the sample using a multistage random sampling technique. This cross-sectional survey was performed using a self-administered questionnaire that measured the constructs of TPB. A structural equation model was used to identify specific factors that most contribute to and predict pharmacists’ intention to provide diabetes care.

**Results:**

The sample included 385 pharmacies, approximately half of them (51.4%) were males and the majority of them (94%) had a bachelor’s degree in pharmaceutical sciences. Intention was significantly correlated with attitude, subjective norm and perceived behavioral control. “Regular screening for complications can improve quality of life for diabetic patients” (β = 1.131) was the most specific factor motivating pharmacists to perform diabetes care, while “some physicians do not appreciate pharmacists’ involvement in diabetic care” was the most specific factor that negatively influenced intention of pharmacists to provide diabetes care (β = 4.283).

**Conclusion:**

Community pharmacists demonstrated a positive attitude, perceived significant approval from others and felt able to intervene in diabetes care. However, lack of physician collaboration was a specific hindering factor for pharmacists’ practice of diabetes care.

**Supplementary Information:**

The online version contains supplementary material available at 10.1186/s12913-022-08788-4.

## Introduction

Diabetes is as a set of metabolic diseases characterized by hyperglycemia, which results from defects in insulin secretion, insulin action, or both [1]. As a rising disease, according to the International Diabetes Federation (IDF 2017), approximately 425 million adults aged 20–79 are diabetic worldwide. This number is estimated to increase to 629 million people in 2045 [2]. Diabetes, if not well controlled, may result in loss of vision, kidney failure, stroke, lower limb amputation and several other long-term consequences that notably affect on quality of life [3].

Regarding the MENA region, 73 million adults (20–79 years old) are living with diabetes according to the latest report of IDF (2021). This figure is estimated to increase to 95 million by 2030 and 136 by 2045. Moving to North Africa, and according to the WHO, the prevalence of diabetes in Egypt in 2014 was 17.9%, the third highest prevalence in the MENA region. In 2019, Egypt occupied the second highest rank among MENA countries with respect to number of adults living with diabetes, with almost 9 million cases [4].

Diabetes is a chronic disease, which is difficult to manage by a single health care provider, as physician’s visits are often short and focus on an acute complaint. Thus, cooperation is necessary between physicians, nurses, dieticians, and pharmacists. Such cooperation will provide patient with diabetes with the adequate care needed [5]. Pharmacist's role in diabetes care is mainly classified into two categories; screening and patient education [6]. This includes setting and monitoring diabetes treatment goals. They can offer training to the patients to use home glucose meter, encourage them to adhere to their medications, stop smoking, and to be assessed annually. Moreover, pharmacists have the opportunity to regularly check patients’ feet, skin, blood pressure, and weight. They should refer them to their physician as needed [7, 8].

**Fig. 1:**
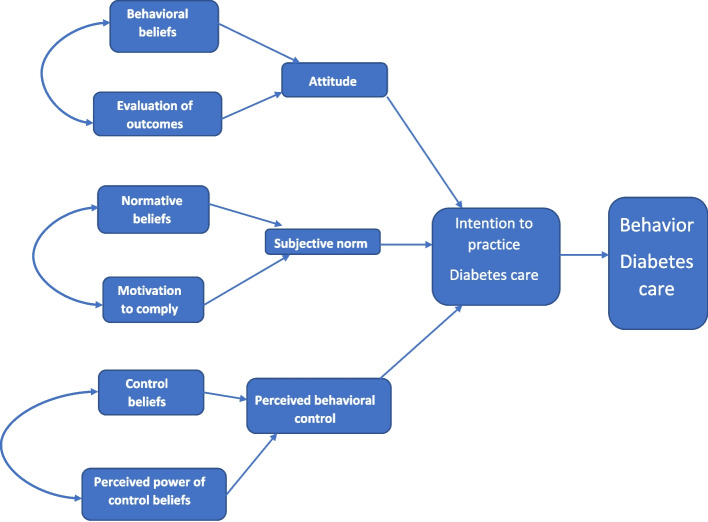
Theory of Planned Behavior

The Theory of Planned Behavior (TPB) (Fig. 1) started as the Theory of Reasoned Action in 1980 to predict an individual's intention to engage in a behavior at a specific time and place. The theory was intended to explain all behaviors over which people have the ability to exert self-control. The key component to this model is behavioral intent; behavioral intentions are influenced by the attitude about the likelihood that the behavior will have the expected outcome and the subjective evaluation of the risks and benefits of that outcome [9].

The theory of Planned Behavior (TPB) (Fig. 1) focuses on individual motivational factors as determinants of a certain behavior. Theory of reasoned action (TRA) suggests that behavioral intention is the most antecedent determinant of behavior and that intention is influenced by attitude towards the behavior and subjective norm about the behavior. TRA assumes that an individual has complete volitional control to carry out a certain behavior. However, this assumption is sometimes unrealistic, since certain behaviors are not under complete volitional control. This led to the proposal of TPB by incorporating a new component, which is perceived behavioral control [10].

Attitude is the first antecedent of intention, it refers to whether an individual like or dislike a certain behavior. It can be estimated directly or indirectly by multiplying one's beliefs regarding outcomes of carrying out a specific behavior (behavioral beliefs) by evaluations of those outcomes. Similarly, subjective norm which refers to social pressure and social norms, can be assessed directly or indirectly by multiplying normative beliefs (whether important referent individuals approve or disapprove of performing the behavior) by motivation to comply with significant referents. Perceived control which refers to the degree to which an individual feels that performing a certain behavior falls under his/her volitional control, is determined directly or indirectly by multiplying control beliefs- the presence or absence of facilitators or barriers- to perform a certain behavior by the perceived power (impact of each control factor to facilitate or inhibit the behavior) of each control factor [11–14].

Many health behaviors and intentions to perform these behaviors were explained by using TPB. These behaviors include smoking cessation, performing exercise, health services consumption, HIV/STD (Human Immunodeficiency Virus /Sexually Transmitted Diseases) prevention, using contraceptive methods, and performing mammography [15].

A randomized controlled trial conducted in Iran using diabetes educational program based on TPB showed that TPB was effective framework, as it helped in improving blood sugar level and adapting retinopathy preventive behaviors [16]. Similar results were shown by a study conducted in China, where quality of life of middle aged stroke patients improved. The intervention of this study was based on the HBM (Health Belief Model) and TPB frameworks [17].

Pharmacists have a vital role in patient care. A systematic review and meta-analysis, showed that pharmacists-led interventions resulted in improvement in inhalation technique in asthma and COPD (Chronic Obstructive Pulmonary Disease) patients. [18]. Moreover, a study conducted in Egypt indicated that pharmacist-led health coaching can enhance breast cancer preventive behaviors [19].

Recently, during the COVID-19 outbreak, community pharmacists had novel roles such as raising awareness regarding personal hygiene, controlling of drug misuse especially vitamin C and other over the counter drugs, as well as providing homecare and telehealth services [20].

Owing to the scarcity of research on the role of the community pharmacist in diabetes care, this study aimed at assessing intention of pharmacist regarding diabetic patients using Theory of Planned Behavior (TPB) as a framework. Hopefully the results in this study would draw the attention of the decision makers in the health sector to the importance of providing pharmacists with the training needed to qualify them to give appropriate counseling. To this point, the current study would be the first to assess community pharmacists’ intention to perform diabetes care using the Theory of Planned Behavior.

## Material and methods

### Sampling

Assuming that 50% of pharmacists had low intention regarding diabetes care and with degree of precision = 5%, the minimum required sample size at the 95% confidence level was 384, which was rounded to 385.

Pharmacies were selected using multistage random sampling where, five health districts in Alexandria with the greatest number of pharmacies were chosen and two neighborhoods were randomly selected from each of the five health districts. Pharmacies were selected randomly from the list of pharmacies in each neighborhood and were obtained from websites [21, 22] by proportion allocation using a systematic random sampling technique.

There was no pharmacist absenteeism. In addition, when pharmacists refused to participate, another pharmacy was chosen.

It was self-administered questionnaire requiring almost 30 min. to complete.

### Study design and settings

A cross sectional study was conducted, participants are encouraged to enroll after explaining the aim of the study. Target population was community pharmacists in Alexandria who have experience at least one year in their field.

#### Inclusion and exclusion criteria

Community pharmacists in Alexandria who had at least one year of experience in their field were included. Any pharmacist who refused participation was excluded, and another pharmacist was included.

The targeted behavior was performing diabetes care practices such as blood glucose monitoring, hypoglycemia management, sick day management, and providing counseling regarding different antidiabetic drugs and diabetes complications.

#### Ethical approval

The approval of our study was attained from the ethical committee. Consent was obtained verbally from all participants.

### Data collection method and tool

A pre-coded and pilot tested self-administered questionnaire [11, 23, 24] (Additional file 1: Appendix 1) was designed and completed within 30 min by the researcher. The tool was also tested for its content validity by two experts in the field of behavioral sciences.

The questionnaire was administered in English and was used to collect the following data:

### Personal characteristics of the studied pharmacists

#### TPB constructs

The scale measuring TPB constructs was designed based on the methodology described by Ajzen [12]. The constructs were measured in two ways: direct and indirect measurements. Responses to each item were scored on a five-point Likert scale ranging from (1) strongly disagree to (5) strongly agree.

Direct measures: Pharmacists’ intention to provide diabetic care was measured by five statements (example: “I intend to provide diabetic patients with information concerning the suitable diet” and “I intend to provide diabetic patients with information on the importance of performing regular exercise”) and was classified into low (5-11), moderate (12-18) and high (19-25). The statements were tested for reliability using Cronbach’s alpha, which was 0.868.

Pharmacists’ attitude towards diabetic care was assessed through six statements (example: “In general, I believe that pharmacists should be involved in giving instructions on use of glucose meters” and “In general, I believe that pharmacists should be involved in advising patients to perform regular check for diabetes complications.” Attitude was classified into; negative (6-13), neutral (14-22) and positive (23-30). The statements were tested for reliability using Cronbach’s alpha, which was 0.901.

Subjective norm was assessed using two statements: “Most people I deal with as a pharmacist encourage my involvement in diabetes care” and “It is expected of me to be involved in diabetes care”. It was classified into; negative (2-4), neutral (5-7) and positive (8-10). The statements were tested for reliability using the Cronbach’s alpha, which was 0.595.

Perceived behavioral control was assessed using two statements: “I am confident that I could identify clients at risk of diabetes mellitus type 2” and “Counseling patients on the drug intake and compliance is easy to me”. It was classified into; low (2-4), moderate (5-7) and high (8-10). The statements were tested for reliability using the Cronbach’s alpha which was 0.671.

Indirect measures-Behavioral beliefs: Respondents were asked to indicate their agreement on 6 outcomes to practicing diabetes care (example: “counseling patients on drug intake and compliance can decrease drug interactions”), and their evaluation of these outcomes (example: In my opinion decreasing drug interactions is (very unimportant, 1 to very important, 5)).

Responses to each pair of items were multiplied together and an indirect measure of attitude (i.e., a belief-based measure of attitude) was subsequently constructed by summing these products across all beliefs. The range of possible scores was 6 to 150.

Normative beliefs: Respondents were asked to indicate the extent to which 5 important referents would approve or disapprove of pharmacists practicing diabetes care (example: Most pharmacists I work with promote the profession of pharmacy through involvement in patient counseling) and the extent to which they were motivated to comply with these referents (example: Generally, I would like to do what other pharmacists do). Each of the two variables was measured using 6 statements rated on a 5-point ranging from strongly disapprove (1) to strongly approve (5). Responses to each pair of items were multiplied together and an indirect measure of subjective norm (i.e., a belief-based measure of subjective norm) was subsequently constructed by summing these products across all referents. The range of possible scores was 6 to 150.

Control beliefs: Respondents indicated the presence or absence of 5 control factors that would hinder practicing diabetes care (example: I feel I do not have enough time to counsel diabetic patients on drug intake and compliance) and the impact of each factor on hindering the behavior (example: Lack of time makes it more difficult for me to counsel patients on drug intake and compliance). Each of the two variables was scored using a 5-point Likert scale ranging from strongly disapprove (5) to strongly approve (1). Responses to each pair of items were multiplied together and an indirect measure of perceived behavioral control (i.e., a belief-based measure of perceived behavioral control) was subsequently constructed by summing these products across all beliefs. The range of possible scores was 5 to 125.

### Statistical analysis

After data was collected, it was revised, coded and entered into the statistical software IBM SPSS version 21. Cronbach's alpha was used to test for reliability of the direct and indirect measures. Descriptive statistics were conducted to describe personal characteristics, while Pearson correlations between the belief-based measures and their corresponding direct measures were examined to determine if the appropriate beliefs were identified and properly measured. Structural equation modeling (SEM) using add-in analysis of moment structure in SPSS – AMOS, was applied to the TPB questionnaire. β-coefficient values calculated from SEM were used to determine the most specific factor to be the predictor.

## Results

### Personal characteristics of the studied pharmacists

The sample included 385 pharmacies with one index per site (one pharmacist per pharmacy), and approximately half of them (51.4%) were males. The average age of the studied pharmacists was 31.10 years (± 9.22 years). The mean years of experience was 7.74 years (± 8.20 years). The majority of the studied pharmacists (94%) had a bachelor’s degree in pharmaceutical sciences, and only 0.8% of them had a master’s degree. Only 3.4% of the studied pharmacists were suffering from diabetes mellitus. Approximately two thirds of the studied pharmacists (64.4%) had not ever worked in a hospital pharmacy, while 6% did and were still working in a hospital pharmacy at the time of the study. More than half (59%) of the studied pharmacists did not attend any training sessions about diabetes care, (Table 1).

**Table 1:** Personal characteristics of the studied community pharmacists

**Pharmacists’ characteristics** (*n* = 385)	**no**	**%**
**Gender**		
Male	198	51.4
Female	187	48.6
**Age in years**		
21- < 30	210	54.5
30 +	175	45.5
Range (21–66)		
Mean ± SD	31.10 ± 9.22	
**Years of experience as a community pharmacist**		
< 5	187	48.6
5 +	198	51.4
Mean ± SD	7.74 ± 8.20	
**Qualification**		
Bachelor	362	94.0
Diploma	15	3.9
Master	3	0.8
PhD	5	1.3
Pharm D	0	0
**Being a diabetic patient**		
No	372	96.6
Yes	13	3.4
**Family history of diabetes mellitus**		
No	175	45.4
Yes, second degree relative	105	27.3
Yes, first degree relative	105	27.3
**Working in a hospital pharmacy**		
No	248	64.4
Yes, in the past	114	29.6
Yes, till now	23	6.0
**Attendance of any training session about diabetes care**		
No	227	59.0
Yes	158	41.0

### Correlations and associations between belief-based measures and direct measures of TPB constructs

The means of all the direct cognitive measures were high (intention = 19.51 ± 4.49, attitude = 23.47 ± 5.39, subjective norm = 7.16 ± 1.75, and perceived behavioral control 7.42 ± 1.69), indicating strong intention, positive attitude, subjective norm and perceived behavioral control of pharmacists towards practicing diabetes care.

Intention was significantly correlated with attitude, subjective norm and perceived behavioral control. The other components of the model were also significantly correlated with each other. Attitude was strongly correlated with intention (0.709), indicating that the more positive attitude the pharmacist has, the more likely he/she is to intend to practice diabetes care. However, attitude was not the only important predictor of pharmacists’ intention, subjective norm and perceived behavioral control had an important impact on pharmacist intention as well. These results lend support to the conceptual framework suggested by the TPB, (Table 2).

**Table 2:** Associations between belief-based measures and direct measures of TPB constructs

	**Correlation coefficient **^**#**^ **Direct measures** ***n* = 385**			
	**Intention**	**Attitude**	**Subjective norm**	**Perceived behavioral control**
**Direct measures**				
Intention^1^	**-**			
Attitude^2^	0.709^**^	**-**		
Subjective norm^3^	0.478^**^	0.478^**^	**-**	
Perceived behavioral control^4^	0.482^**^	0.497^**^	0.445^**^	**-**
**Belief-based measures**				
Behavioral beliefs^5^	0.678**	0.705**	**-**	**-**
Normative beliefs^6^	0.447^**^	**-**	0.572^**^	**-**
Control beliefs^7^	-0.060	**-**	**-**	-0.185^**^

^**^Correlation matrix is significant at the 0.01 level (2-tailed)

^#^Pearson Correlation

^1^5≤ Intention≤25

^2^6≤ Attitude ≤30

^3^2≤ Subjective norm ≤10

^4^2≤ Perceived behavioral control ≤10

^5^6≤ Behavioral beliefs ≤30

^6^6≤ Normative beliefs ≤30

^7^5≤ Control beliefs ≤25

The mean behavioral belief (23.89 ± 4.39) and normative belief (20.46 ± 4.35) scores were high reflecting positive pharmacists’ attitude towards diabetes care and strong positive social pressure to perform this care, respectively. Meanwhile, the mean control belief score (14.26 ± 3.01) was somewhat lower than the other belief-based scores, suggesting that it was to some extent difficult for pharmacists to provide diabetes care.

### Influencers and predictors of pharmacists’ intention

**Fig. 2:**
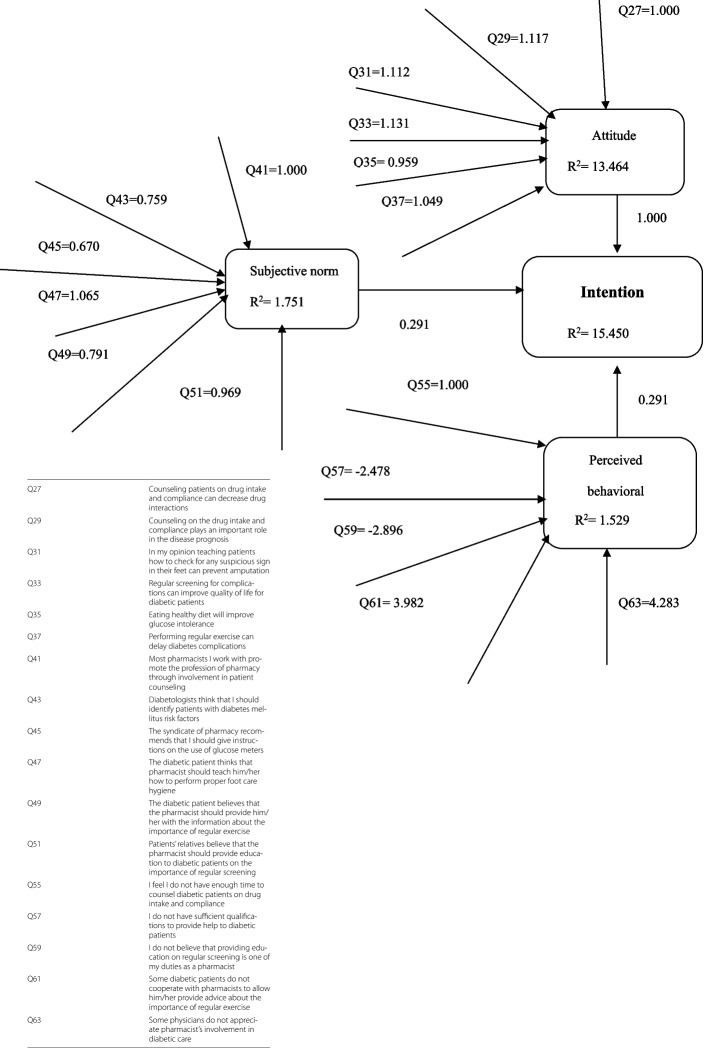
Structural equation model of pharmacists’ intention to perform diabetes care practices

Attitude was the most important predictor of intention (standardized estimate = 1.000), followed by subjective norm and perceived behavioral control (standardized estimate = 0.291). All of these three components contributed to 15.5% of the pharmacists’ intention to provide diabetes care. Interestingly, attitude alone was able to explain 13.5% of the variance in intention, while subjective norms and perceived behavioral control explained 1.75% and 1.53% of the variance in intention respectively, (Fig. 2).

Structural equation modeling (SEM) further revealed which beliefs played an important role in influencing pharmacists’ intention to provide diabetes care. The beliefs underlying attitudes that were most strongly connected to intention were "regular screening for complications can improve quality of life for diabetic patients" (standardized estimate = 1.131), and “counseling on the drug intake and compliance plays an important role in the disease prognosis” (standardized estimate = 1.117). This indicated that pharmacists were first and foremost looking at preventing or at least delaying diabetes complications Fig. 2.

As shown in Fig. 2, "diabetic patients" were perceived by pharmacists as the most influential referents (standardized estimate = 1.065) followed by “patients’ relatives” (standardized estimate = 0.969). Therefore, the greater the perceived social support from these people to provide diabetes care, the more likely pharmacists were to intend to provide this care. On the other hand, " Some physicians do not appreciate pharmacist’s involvement in diabetic care”" was the most specific factor that negatively influenced intention of pharmacists to perform certain diabetes care practices (standardized estimate = 4.283).

## Discussion

This research aimed to identify the factors affecting pharmacists’ intention to perform diabetes care. The results showed that pharmacists generally held high intentions to practice diabetes care. They had fairly positive attitudes towards diabetes care, perceived positive social pressure to do so and felt in control of practicing diabetes care. Correlational analyses showed that attitude was strongly associated with intention followed by perceived behavioral control then subjective norm. These findings highlight the influential role of attitude in motivating pharmacists to perform diabetes care. Hence, the descending order of these relationships should be considered when designing health education programs to enhance pharmacists’ intention to practice diabetes care. Similarly, a study conducted in Kuwait showed that pharmacists had generally positive attitudes regarding management of diabetes [25].

Moreover, a cross-sectional study conducted in Egypt to explain community pharmacists’ intention to provide pharmaceutical care (PC) services, showed that the PCI (Pharmaceutical Care Intention) scale is a parsimonious, theory-driven instrument with acceptable construct validity and reliability to examine factors associated with community pharmacists’ intention to provide PC [26]. Another cross-sectional, self-administered survey was completed by a random sample of community pharmacists in Alexandria, Egypt to predict Egyptian community pharmacists’ counseling on oral contraceptives while utilizing a theoretical framework guided by the Theory of Planned Behavior (TPB). This study showed that the TPB appears to help predict pharmacists’ OC (oral contraceptives) counseling. It also indicated that the pharmacists mainly talked to women about the importance of taking the OC at the same time every day, and that female pharmacists were more welcomed than males to counsel women [27]. Additionally, a cross-sectional questionnaire based on the theory of planned behavior (TPB), was conducted in Saudi Arabia to predict the pharmacists’ willingness in this nation to commit to providing MTM (Medication therapy management) services there. This study revealed that the majority of pharmacist had strong intention, positive attitude and felt that they had control towards providing MTM services. Also, pharmacists surveyed believed that people who are important to them would encourage them to provide MTM services [28].

On the other hand, a cross-sectional study conducted in Maryland revealed that perceived ease of counseling and subjective norm were the socio-cognitive variables significantly predicting community pharmacists’ intention to provide pediatric asthma counselling [29]. Meanwhile, self-efficacy seemed to be the most important factor affecting public intention to visit the dentist followed by attitude and subjective norm in the described order [30]. According to Fishbein, the relative weights of the model components depend upon the behavior and the population under investigation. Some behaviors are mainly under attitudinal control, others are mostly under normative control. The same behavior could be mainly affected by attitude in one population but by normative beliefs in another population [31].

In this research, the amount of variance in pharmacists’ intention explained by the components of the model (15.5%) was somewhat modest. Perhaps, the addition of past behavior would have increased the predictive power of the model. Ajzen postulates that intention to perform a behavior can be affected by prior behavioral experience [32].

By examining the relationship between belief-based measures and intention, it was found that pharmacists’ intention to perform diabetes care was significantly affected by behavioral and normative beliefs, while the influence of control beliefs on pharmacists’ intention was not significant. It appears that the studied control factors did not capture all the important factors that control pharmacists’ diabetes care practices well. Elicitation interviews should be used to find out more influencing control factors in future research. Moreover, control beliefs demonstrated in the current study showed a weak correlation with perceived behavioral control. One plausible explanation is that the direct measure of perceived behavioral control used in this study assessed only pharmacists’ confidence in their ability to perform diabetes care (self-efficacy) and did not inquire about their controllability over the behavior when the control factors identified in the structured interviews focused mainly on external aspects of control (controllability) on the expense of internal aspects. Perhaps in future research, it would be better to divide the direct measure of perceived behavioral control into direct perceived control and direct self-efficacy.

Specific factors influencing pharmacists’ intention to practice diabetes care were pharmacists’ beliefs that “regular screening for complications can improve quality of life for diabetic patients” and “counseling on the drug intake and compliance plays an important role in the disease prognosis”. Similarly, a qualitative TPB-based study conducted in Quebec, Canada on pharmacists’ interventions with chronic kidney disease (CKD) patients, revealed that pharmacists perceived that they can prevent an acute renal failure leading to hospitalization [33].

The results of the present study indicated that “diabetic patients” were the most significant referents influencing pharmacists’ intention to perform diabetes care. Other referents including patients’ relatives, diabetologists, syndicate of pharmacy and other pharmacists showed less influence. This finding implies that diabetic patients’ attitude towards pharmacists and patients showing the will and skill of attending to pharmacists’ advice would motivate pharmacists to engage in diabetes care. The aforementioned qualitative TPB based study conducted in Canada investigated the influence of normative beliefs of community pharmacists on interventions in chronic kidney disease (CKD) patients. Pharmacists perceived CKD patients as eager to seek professional advice and reported that the great majority of them would agree to the pharmacist intervening. They perceived young physicians to be more favorable to pharmacists intervening and perceived that their colleagues generally agreed with their interventions and that their interventions were appreciated by nurses and hospital pharmacists [33]. Among the investigated referents, the present study showed that diabetic patients were the most influential group with regard to pharmacists practicing diabetes care. This finding suggests that the pharmacist–patient relationship plays a key role in diabetes care practice. This topic may require further investigation.

The most specific demotivating factor was “Some physicians do not appreciate pharmacist’s involvement in diabetic care”, followed by “Some diabetic patients do not cooperate with pharmacists to allow him/her provide advice about the importance of regular exercise”. On the other hand, the former Canadian study concluded that the first common barrier to pharmacists’ intervention was lack of time and the second common barrier was lack of pharmacists’ specific and advanced knowledge in CKD [33]. This discrepancy may be due to the difference in healthcare structure between both countries.

The present study underscores the importance of the pharmacist-physician–patient triad in supporting pharmacists’ intention to intervene in diabetes care. For community pharmacists to perform a proactive broader clinical role, inter-professional collaboration will need to be encouraged. Channels and links between pharmacists and physicians have to be created and fostered. The clinical role of pharmacists and their practice of systematic pharmaceutical interventions should be incorporated into the training programs of pharmacists and physicians as well.

The current study was able to uncover some of the factors associated with pharmacists’ intention regarding diabetes care using the theory of planned behavior (TPB), which was shown to be an appropriate conceptual framework that served well in the fulfillment of this goal. However, this study had a few limitations. First, the facilitators of providing diabetes care were not measured and only barriers were assessed. Second, the present study did not assess pharmacists’ prior behavior of providing diabetes care. The addition of a measure of prior behavior would have probably added to the amount of variance in pharmacists’ intention. Then, there was no representation for all districts in Alexandria. Picking places with "greatest number of pharmacies” and excluding places with a smaller number of pharmacies affected our findings (those may be more involved with patient care). Last, the current study relied on literature review and on the authors' experience in designing the questionnaires; however, conducting an in-depth, open-ended elicitation interview prior to formulation of the questionnaire would have identified more relevant diabetes care-related control beliefs.

## Conclusion

Community pharmacists demonstrated a positive attitude, perceived significant approval from others and felt able to intervene in diabetes care. However, lack of physician collaboration was a specific hindering factor for pharmacists’ practice of diabetes care. Believing that regular screening for complications can improve quality of life of diabetic patients was a specific factor that enhanced pharmacists' attitude towards diabetes care. Diabetic patients were the most important supporters to engage pharmacists in diabetes care. An understanding of these motivational factors and barriers is invaluable in planning for a broader clinical role of pharmacists in diabetes care.

## Supplementary Information

**Additional file 1.**

## Data Availability

Data and questionnaires are available with the corresponding author upon request. The datasets used and/or analyzed during the current study available from the corresponding author on reasonable request.

## Abbreviations

CKD: Chronic Kidney Disease
COPD: Chronic Obstructive Pulmonary Disease
HBM: Health Belief Model
HIV/STD: Human Immunodeficiency Virus /Sexually Transmitted Diseases
IDF: International Diabetes Federation
MENA: Middle East and North Africa
MTM: Medication therapy management
OC: oral contraceptives
PC: Pharmaceutical care
PCI: Pharmaceutical Care Intention
SEM: Structural equation modeling
TPB: Theory of Planned Behavior
TRA: Theory of reasoned action
WHO: World health organization
