# Supplementary material for: Applying the Theory of Planned Behavior to predict community pharmacists’ intention to provide diabetes care
Source: BMC Health Serv Res. 2022 Dec 5;22:1479. doi: 10.1186/s12913-022-08788-4 (PMC9721017; doi:10.1186/s12913-022-08788-4)
Supplement: Supplementary file 1 — Additional file 1. [file 12913_2022_8788_MOESM1_ESM.docx]

**Appendix 1**

1-Age

2-Gender

3-Years of experience

4-Location of the pharmacy

5-Opening days of the pharmacy

6-Working hours of the pharmacy

7-Qualification: Bachelor- Diploma- Master- PhD

8-Are you a diabetic patient

1- No

2- Yes

9-Does any of your family members suffer from diabetes mellitus?

1. No
2. Yes, second degree relative
3. Yes, first degree relative

10- Have you ever worked in a hospital pharmacy?

1-No

2-Yes, in the past

3- Yes, till now

11- Did you attend any training session about diabetes care?

1- No

2- Yes

12-Mark the available anti diabetic medications:

(You can choose more than one answer)

Insulin – Metformin- Sulphonylureas – Others

13- How often are they available?

Consistent- sometimes- rarely

14-Mark the available monitoring materials:

(You can choose more than one answer)

Blood testing strips – Glucose meters- Others

15- How often are they available?

Consistent- sometimes- rarely

- The following statements represent the intention of pharmacists to perform some diabetic care practices in the future:

| As a pharmacist I intend to: | Strongly disagree  (1) | Disagree  (2) | Not sure  (3) | Agree  (4) | Strongly agree  (5) |
| --- | --- | --- | --- | --- | --- |
| 16-Counsel diabetic patients on drug intake and compliance. |  |  |  |  |  |
| 17-Give diabetic patients instructions on the use of glucose meters. |  |  |  |  |  |
| 18- Provide diabetic patients education on regular screening for complications. |  |  |  |  |  |
| 19-Provide diabetic patients information concerning the suitable diet. |  |  |  |  |  |
| 20-Provide diabetic patients information on the importance of performing regular exercise. |  |  |  |  |  |

| Below are some statements about diabetes. Each numbered statement finishes the sentence **“In general, I believe that pharmacist should be involved in ...”** You may believe that a statement is true for one person but not for another person or may be true one time but not be true another time. Mark the answer that you believe is true most of the time or is true for most people. Place a check mark in the box below the word or phrase that is closest to your opinion about each statement. It is important that you answer every statement. | | | | | |
| --- | --- | --- | --- | --- | --- |
|  | Strongly disagree  (1) | Disagree  (2) | Not sure  (3) | Agree  (4) | Strongly agree  (5) |
| 21-... giving instruction on use of glucose meters. |  |  |  |  |  |
| 22-...advising patients to regular check for diabetes complications. |  |  |  |  |  |
| 23-... teaching diabetic patients how to prepare healthy diet. |  |  |  |  |  |
| 24-...providing diabetic patients with the benefits of performing regular exercise. |  |  |  |  |  |
| 25-... counseling on proper diabetic foot care techniques. |  |  |  |  |  |
| 26-…counseling diabetic patients on drug intake and compliance. |  |  |  |  |  |

| The following statements represent the consequences of performing certain diabetic care practices and their importance: | | | | | |
| --- | --- | --- | --- | --- | --- |
| 27-Counseling patients on drug intake and compliance can decrease drug interactions. | Strongly disagree  (1) | Disagree  (2) | Not sure  (3) | Agree  (4) | Strongly agree  (5) |
| 28-In my opinion decreasing drug interactions is | Very unimportant  (1) | Unimportant  (2) | Neutral  (3) | Important  (4) | Very important  (5) |
| 29-Counseling on the drug intake and compliance plays an important role in the disease prognosis | Strongly disagree  (1) | Disagree  (2) | Not sure  (3) | Agree  (4) | Strongly agree  (5) |
| 30- In my opinion reaching a good disease prognosis is | Very unimportant  (1) | Unimportant  (2) | Neutral  (3) | Important  (4) | Very important  (5) |
| 31-In my opinion teaching patients how to check for any suspicious sign in their feet can prevent amputation | Strongly disagree  (1) | Disagree  (2) | Not sure  (3) | Agree  (4) | Strongly agree  (5) |
| 32-I believe that preventing amputation is | Very unimportant  (1) | Unimportant  (2) | Neutral  (3) | Important  (4) | Very important  (5) |
| 33-Regular screening for complications can improve quality of life for diabetic patients | Strongly disagree  (1) | Disagree  (2) | Not sure  (3) | Agree  (4) | Strongly agree  (5) |
| 34-Improving quality of life for diabetic patients is | Very unimportant  (1) | Unimportant  (2) | Neutral  (3) | Important  (4) | Very important  (5) |
| 35-Eating healthy diet will improve glucose intolerance | Strongly disagree  (1) | Disagree  (2) | Not sure  (3) | Agree  (4) | Strongly agree  (5) |
| 36-I believe that improving glucose intolerance is | Very unimportant  (1) | Unimportant  (2) | Neutral  (3) | Important  (4) | Very important  (5) |
| 37-Performing regular exercise can delay diabetes complications | Strongly disagree  (1) | Disagree  (2) | Not sure  (3) | Agree  (4) | Strongly agree  (5) |
| 38-Delaying diabetes complications is | Very unimportant  (1) | Unimportant  (2) | Neutral  (3) | Important  (4) | Very important  (5) |

| The following statements measure the overall social pressure on the pharmacists: | Strongly disapprove  (1) | Disapprove  (2) | Not sure  (3) | Approve  (4) | Strongly approve  (5) |
| --- | --- | --- | --- | --- | --- |
| 39-Most people I deal with as a pharmacist encourage my involvement in diabetes care. |  |  |  |  |  |
| 40-It is expected of me to be involved in diabetes care. |  |  |  |  |  |

| The following statements demonstrate how salient people/institutions perceive the pharmacist’s involvement in diabetes care: | Strongly disapprove  (1) | Disapprove  (2) | Not sure  (3) | Approve    (4) | Strongly approve  (5) |
| --- | --- | --- | --- | --- | --- |
| 41-Most pharmacists I work with promote the profession of pharmacy through involvement in patient counseling |  |  |  |  |  |
| 42-Generally I would like to do what other pharmacists do |  |  |  |  |  |
| 43-Diabetologists think that I should identify patients with diabetes mellitus risk factors |  |  |  |  |  |
| 44-What diabetologists think I should do matters to me |  |  |  |  |  |
| 45-The syndicate of pharmacy recommends that I should give instructions on the use of glucose meters |  |  |  |  |  |
| 46-What the syndicate of pharmacy recommends is important to me |  |  |  |  |  |
| 47-The diabetic patient thinks that pharmacist should teach him/her how to perform proper foot care hygiene |  |  |  |  |  |
| 48-Responding to my patient needs is important to me |  |  |  |  |  |
| 49-The diabetic patient believes that the pharmacist should provide him/her with the information about the importance of regular exercise |  |  |  |  |  |
| 50-Responding to my patient needs is important to me |  |  |  |  |  |
| 51- Patients’ relatives believe that the pharmacist should provide education to diabetic patients on the importance of regular screening. |  |  |  |  |  |
| 52- What my patients’ relatives believe I should do matters to me |  |  |  |  |  |

| These statements show to what extent the previously mentioned behaviors are under the control of pharmacists: | Strongly disapprove  (1) | Disapprove  (2) | Not sure  (3) | Approve  (4) | Strongly approve  (5) |
| --- | --- | --- | --- | --- | --- |
| 53-I am confident that I could identify clients at risk of diabetes mellitus type 2 |  |  |  |  |  |
| 54-Counseling patients on the drug intake and compliance is easy to me |  |  |  |  |  |

| The following statements indicate the barriers and facilitators for performing some diabetes care practices, and their effect: | Strongly disapprove  (5) | Disapprove  (4) | Not sure  (3) | Approve  (2) | Strongly approve  (1) |
| --- | --- | --- | --- | --- | --- |
| 55-I feel I do not have enough time to counsel diabetic patients on drug intake and compliance |  |  |  |  |  |
| 56-Lack of time make it more difficult for me to counsel patients on drug intake and compliance |  |  |  |  |  |
| 57-I do not have sufficient qualifications to provide help to diabetic patients |  |  |  |  |  |
| 58-Absence of the sufficient qualifications impedes me to help diabetic patients |  |  |  |  |  |
| 59-I do not believe that providing education on regular screening is one of my duties as a pharmacist |  |  |  |  |  |
| 60-Believing that it is not my duty to provide education on regular screening impedes me from doing so |  |  |  |  |  |
| 61-Some diabetic patients do not cooperate with pharmacists to allow him/her provide advice about the importance of regular exercise |  |  |  |  |  |
| 62-Non-cooperative diabetic patients make it more difficult for me to provide advice about the importance of regular exercise |  |  |  |  |  |
| 63-Some physicians do not appreciate pharmacist’s involvement in diabetic care |  |  |  |  |  |
| 64-Some physicians’ non-appreciation of pharmacist’s involvement discourages me from helping diabetic patients |  |  |  |  |  |

**Appendix 2**

| **Reporting guideline** | **Questions to address in the manuscript** |
| --- | --- |
| **Introduction** | |
| 1. Provide a rationale for using a survey. | The surveys are ideal tools to collect data regarding behavior and attitudes |
| **Method** | |
| 1. Describe how the survey was created or adapted from existing survey(s). | - Pre-coded and pilot tested self-administered questionnaire ^(11, 23, 24)^ (appendix1) was designed and completed by researcher. - The scale measuring TPB constructs was designed based on the methodology described by Ajzen. ^(11)^ |
| 1. Describe how the survey was pretested prior to full implementation. | A pilot study of 10% of sample was conducted to test questionnaire and it was modified according to the results. |
| 1. Describe the final survey instrument, including how and when it was administered. | The final questionnaire used to collect data about the following:  Personal characteristics of the studied pharmacists (Q1-Q15)   - Intention (Q16-Q20) - Direct measure of attitude (Q21-Q26) - Indirect measure of attitude through multiplying behavioral beliefs (Q27, Q29, Q31, Q33, Q35, and Q37) by evaluation of outcomes (Q28, Q30, Q32, Q34, Q36, and Q38) - Direct measure of subjective norm (Q39, and Q40) - Indirect measure of subjective norm through multiplying normative beliefs (Q41, Q43 Q45, Q47, Q49, and Q51) by motivation to comply (Q42, Q44, Q46, Q48, Q50, and Q52) - Direct measure of perceived behavioral control (Q53 and Q54) - Indirect measure of perceived behavioral control through multiplying control beliefs (Q55, Q57, Q59, Q61, Q63) by perceived power of control beliefs (Q56, Q58, Q60, Q62, Q64) - It was a self-administered questionnaire requiring almost 30 minutes to complete. It was administered to community pharmacists during their work to be completed in their free time and resubmitted. |
| **Results** | |
| 1. Describe the respondents, response rate, and how nonresponse bias was assessed. | There was no pharmacist absenteeism.  In addition, when pharmacists refused to participate, another pharmacy was chosen.  Approximately double the number of the sample were approached (385*2=770) in order to compensate for refusal, half of them agreed to participate (385). |
| 1. Describe how score reliability and validity were assessed | - Reliability was used to assess internal consistency of the different scales. It was calculated using chronbach’s alpha. - The tool was also tested for its content validity by two experts in the field of behavioral sciences. |
